# On-chip optical mode exchange using tapered directional coupler

**DOI:** 10.1038/srep16072

**Published:** 2015-11-04

**Authors:** Zhonglai Zhang, Xiao Hu, Jian Wang

**Affiliations:** 1 Wuhan National Laboratory for Optoelectronics, School of Optical and Electronic Information, Huazhong University of Science and Technology, Wuhan 430074, Hubei, China

## Abstract

We present an on-chip optical mode exchange between two multiplexed modes by using tapered directional couplers on silicon-on-insulator platform. The device consisting of mode multiplexing and mode exchange is compact with relatively large fabrication error tolerance. The simulation results show efficient higher order mode excitation and mode exchange. A low excess loss less than 0.5 dB and high extinction ratio larger than 15 dB over 10 nm wavelength range from 1535 to 1545 nm are achieved.

On-chip optical interconnect is a promising technique to satisfy the exponentially increasing demand of bandwidth for future massively-parallel chip multiprocessors^1^. Several techniques have been employed to extend the capacity of optical interconnections. Among them, wavelength-division multiplexing (WDM) is a straightforward way to expand the capacity with multiple wavelengths and has been widely used in long-haul optical communication systems. However, the requirement of multiple laser sources with different wavelengths could be expensive and complicated for on-chip optical interconnection applications. Space-division multiplexing (SDM), which only employs a single wavelength carrier, is another simple way and has been demonstrated by employing multi-core or few-mode fibers^2^,^3^. Mode-division multiplexing (MDM) is a kind of SDM technique which could provide an alternative approach to increasing the link capacity of optical interconnects. The key challenge of an on-chip MDM system is the efficient mode (de)multiplexer. Several kinds of (de)multiplexer have been proposed. The designs based on Y-junctions^4^,^5^,^6^,^7^, multimode interferometer^8^,^9^ and adiabatic couplers^10^ mainly (de)multiplex two channels. In the recent years, schemes using asymmetrical directional couplers (ADC) have been proposed^11^,^12^ and 8-channel hybrid (de)multiplexing combing MDM and polarization-division multiplexing (PDM) has been demonstrated^12^. The ADC can be easily fabricated on silicon-on-insulator (SOI) platform. In order to improve the fabrication tolerance of the ADC, a tapered coupling region is introduced into the ADC^13^.

Very recently, on-chip MDM technology has attached increasing interest. Beyond basic functions such as (de)multiplexer, a laudable goal would be to develop data traffic grooming functions in on-chip MDM systems. Data traffic grooming is considered to be an attractive technique for enhancing the efficiency and flexibility of networks^14^. Lots of data traffic grooming functions have been well studied in WDM systems. Among these functions, data exchange, also known as wavelength exchange/interchange in the wavelength domain^15^,^16^,^17^,^18^,^19^,^20^, is an important technique which can efficiently utilize network resources and facilitate superior network performance. In this scenario, one might also expect to implement data exchange in the mode domain in an on-chip MDM system, i.e. on-chip optical mode exchange^21^.

In this paper, we present an optical exchange function in the mode domain based on tapered directional couplers on SOI platform. We calculate the mode properties of the SOI based nanowires and numerically study the light propagation for optical mode exchange by three dimensional finite difference time domain (3D FDTD) simulations.

## Results

### Structure of the tapered directional coupler

**Figure 1:**
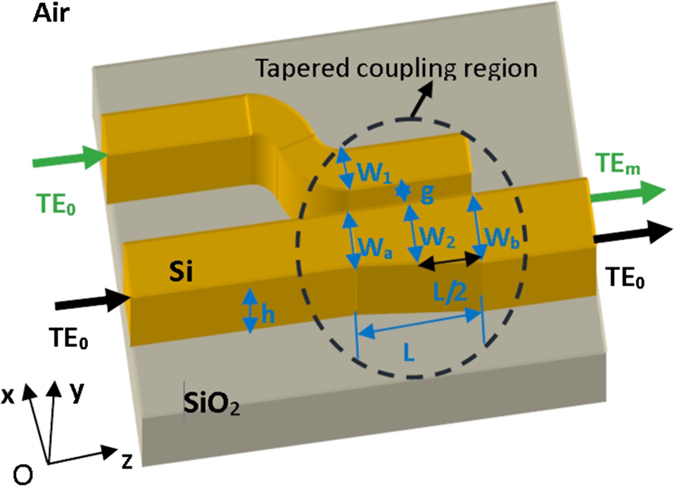
Schematic structure of a mode (de)multiplexer based on a tapered directional coupler.

Figure 1 illustrates the structure of the tapered directional coupler, which couples light from a narrow silicon access waveguide (waveguide width *w*_1_) to a tapered wide multimode bus waveguide (waveguide width from *w*_a_ to *w*_b_ with center width of *w*_2_).

The fundamental mode TE_0_ in the access waveguide is coupled into the multimode bus waveguide through the tapered coupling region and converted to higher order mode TE_m_ (m = 1, 2, 3…). The coupling length is *L* and the gap between the two waveguide is *g*. When *w*_a_ = *w*_b_ = *w*_2_, the structure is a conventional directional coupler, and high efficiency TE_0_-TE_m_ coupling occurs when a phase matching condition (*n*_eff0_ = *n*_effm_, where *n*_eff0_ is the effective index of the fundamental mode in the access waveguide and *n*_effm_ is the *m*^th^ higher order mode in the multimode bus waveguide) is satisfied. However, a large fabrication error of the access waveguide can easily break the phase matching condition in the conventional directional coupler.

### Characterization of mode properties

**Figure 2:**
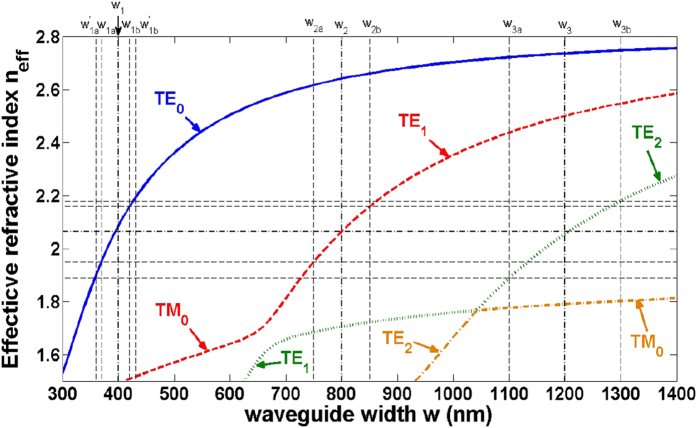
Effective indices of the TE_0_, TE_1_, TE_2_ and TM_0_ modes of an air-cladded SOI waveguide as a function of the waveguide width w for a waveguide height h = 220 nm.

Figure 2 shows the calculated effective refractive indices of the guided-modes in an SOI nanowire with different waveguide width. It can be seen that the slope of the effective refractive index of the TE_0_ mode versus waveguide width is larger than other modes, so the fabrication error induced effective refractive index deviation of TE_0_ mode is also larger than other modes, which means the phase matching condition is more easily to be broken. A tapered wide bus waveguide in the coupling region can relax the limitation. For the two widths *w*_2a_ (*w*_3a_) and *w*_2b_ (*w*_3b_) of the wide tapered waveguide, the corresponding widths of the narrow waveguide which satisfy the phase matching condition (*n*_eff0_ (w_1_) = *n*_effm_ (w_m_)) are *w*_1a_ (*w*_1a_′) and *w*_1b_ (*w*_1b_′), respectively, as indicated in Fig. 2. Consequently, tapering the wide waveguide from *w*_2a_ (*w*_3a_) to *w*_2b_ (*w*_3b_) will result in a deviation tolerance between *w*_1a_ (*w*_1a_′) and *w*_1b_ (*w*_1b_′) for the narrow waveguide, within which a phase matching position can always be found along the taper. One thing should be noted is that *w*_1a_ (*w*_1a_′) should not be too close to the width where the TE_1_ (TE_2_) and TM_0_ modes are hybridized (~660 nm for TE_1_ and ~1040 nm for TE_2_ in Fig. 2).

**Figure 3:**
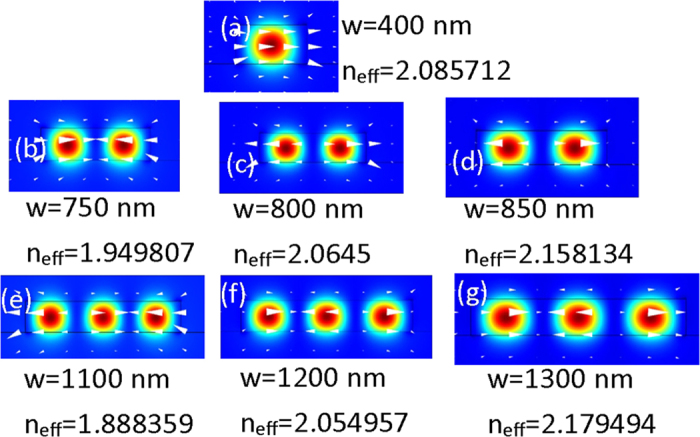
Mode distribution and effective refractive index of (**a**) TE_0_ mode, (**b–d**) TE1 mode and (**e–g**) TE2 mode.

The mode distribution and effective refractive index of the TE_0_, TE_1_ and TE_2_ modes in the silicon nanowire are displayed in Fig. 3(a–g). As shown in Fig. 3(c,f), the effective refractive index of TE_1_ mode with the waveguide width of 800 nm and the effective refractive index of TE_2_ mode with the waveguide width of 1200 nm are nearly equal to the effective refractive index of TE_0_ mode with the waveguide width of 400 nm, which means the phase matching condition can be satisfied.

### Configuration of mode exchange

**Figure 4:**
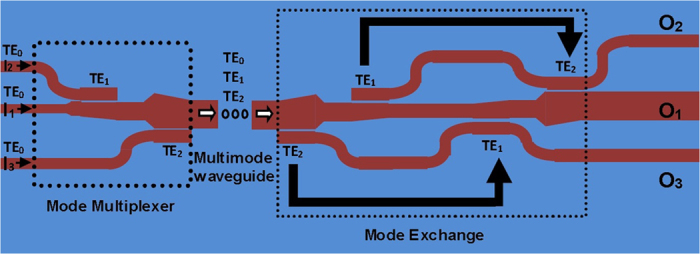
Schematic configuration of optical mode exchange.

The proposed mode exchange configuration is depicted in Fig. 4. The left part in the dashed rectangle is a mode multiplexer. The TE_0_ mode launched in the input port 1 (I_1_) propagates directly in the wide multimode bus waveguide without any change. The TE_0_ modes launched in the input port 2 (I_2_) and input port 3 (I_3_) are coupled into the multimode bus waveguide and converted into the high-order TE_1_ mode and TE_2_ mode by tapered directional couplers, respectively. The three multiplexed modes carrying different data information propagate through the wide multimode bus waveguide simultaneously. The right part in the dashed rectangle accomplishes the mode exchange function. On one hand, TE_2_ mode is coupled into the lower narrow access waveguide as TE_0_ mode and then coupled back into the multimode bus waveguide as TE_1_ mode, i.e. mode conversion from TE_2_ to TE_1_ in the multimode bus waveguide. On the other hand, TE_1_ mode is coupled into the upper narrow access waveguide as TE_0_ mode and then coupled back into the multimode bus waveguide as TE_2_ mode, i.e. mode conversion from TE_1_ to TE_2_ in the multimode bus waveguide. In this way, mode exchange function between the TE_1_ mode and TE_2_ mode can be realized. Meanwhile, the data information carried by the two modes is also exchanged. Mode coupling in the mode exchange part is also achieved by tapered directional couplers.

### Mode exchange results

**Figure 5:**
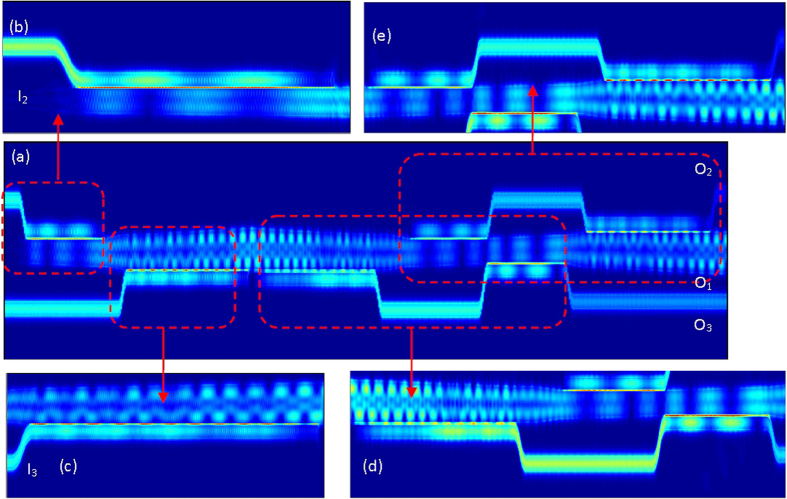
3D FDTD simulation results of light propagation for optical mode multiplexing and mode exchange.

Figure 5 depicts the light propagation simulation results. Shown in Fig. 5(a) is the overall view of the light propagation with mode exchange. TE_0_ mode is launched into both I_2_ and I_3_, leading to the simultaneous excitation of both TE_1_ and TE_2_ modes in the multimode bus waveguide. Shown in Fig. 5(b,c) are the zoomed in views of TE_1_ mode excitation and TE_2_ mode excitation by the launched TE_0_ mode. In this way, both TE_1_ mode and TE_2_ mode exist and propagate in the multimode bus waveguide and mode multiplexing is achieved. The TE_1_ (TE_2_) mode is then coupled into an access waveguide (TE_0_ mode) which is further coupled back into the multimode bus waveguide as the TE_2_ (TE_1_) mode. Shown in Fig. 5(d) is the zoomed in view of TE_2_-TE_0_-TE_1_ mode conversion process. Shown in Fig. 5(e) is the zoomed in view of TE_1_-TE_0_-TE_2_ mode conversion process. As a consequence, mode exchange between TE_1_ and TE_2_ modes (TE_1_ ↔ TE_2_) is implemented.

**Figure 6:**
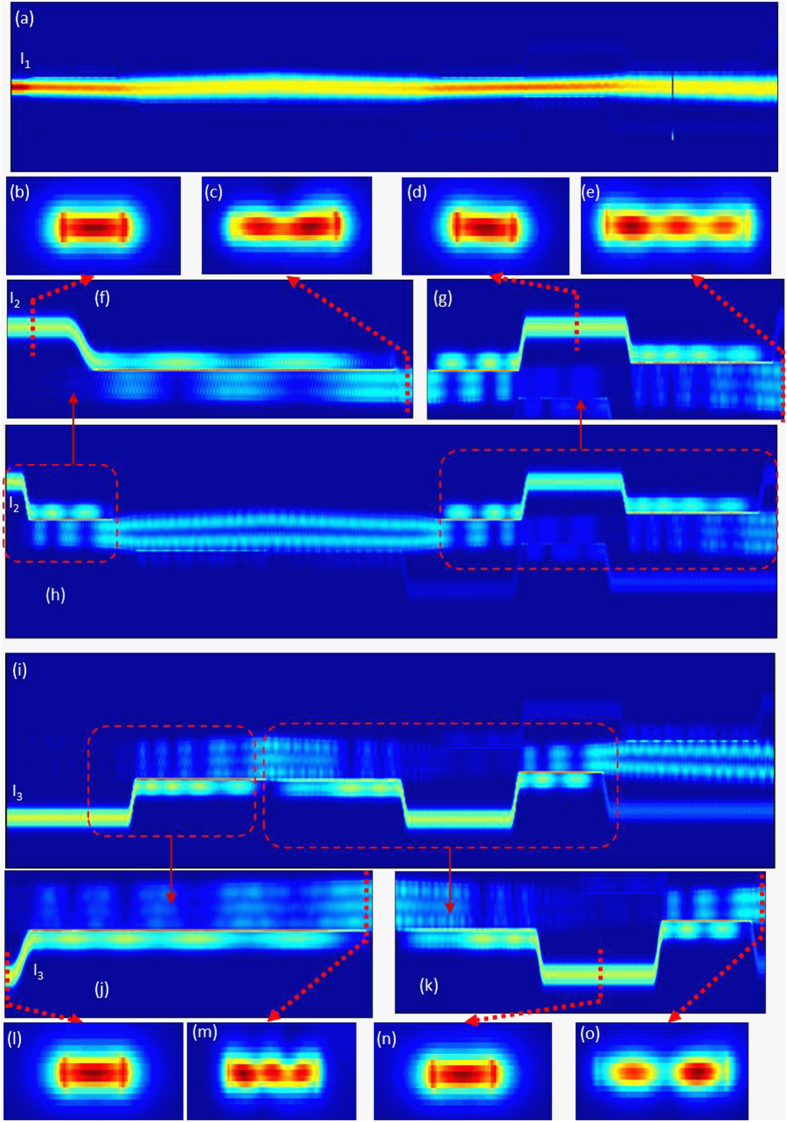
3D FDTD simulation results of light propagation when only (**a**) I_1_ port, (**b–h**) I_2_ port, or (**i–o**) I_3_ port is launched by TE_0_ mode.

In order to clearly show the mode exchange process, we further simulate the light propagation with field monitors placed in the waveguide cross section when only one input port is launched by TE_0_ mode. Figure 6(a) shows the case when only I_1_ port is launched by TE_0_ mode. It can be seen that the TE_0_ mode propagates directly in the multimode bus waveguide. Figure 6(b–h) show the case when only I_2_ port is launched by TE_0_ mode. The whole mode evolution process from TE_0_-TE_1_-TE_0_-TE_2_ is depicted in Fig. 6(h), implying the mode conversion from TE_1_ to TE_2_ in the multimode bus waveguide. Shown in Fig. 6(f) is the zoomed in view of the TE_1_ mode excitation by the input TE_0_ mode. Figure 6(b,c) are the corresponding field profiles of TE_0_ mode and TE_1_ mode monitored in the waveguide cross section. Shown in Fig. 6(g) is the zoomed in view of back conversion from TE_1_ mode to TE_0_ mode and its further conversion to TE_2_ mode. Figure 6(d,e) are the corresponding field profiles of TE_0_ mode and TE_2_ mode monitored in the waveguide cross section. Figure 6(i–o) show the case when only I_3_ port is launched by TE_0_ mode. The whole mode evolution process from TE_0_-TE_2_-TE_0_-TE_1_ is depicted in Fig. 6(i), implying the mode conversion from TE_2_ to TE_1_ in the multimode bus waveguide. Shown in Fig. 6(j) is the zoomed in view of the TE_2_ mode excitation by the input TE_0_ mode. Figure 6(l,m) are the corresponding field profiles of TE_0_ mode and TE_2_ mode monitored in the waveguide cross section. Shown in Fig. 6(k) is the zoomed in view of back conversion from TE_2_ mode to TE_0_ mode and its further conversion to TE_1_ mode. Figure 6(n,o) are the corresponding field profiles of TE_0_ mode and TE_1_ mode monitored in the waveguide cross section. According to the mode conversion from TE_1_ to TE_2_ in the multimode bus waveguide shown in Fig. 6(b,h) and the mode conversion from TE_2_ to TE_1_ in the multimode bus waveguide shown in Fig. 6(I–o), one can expect the mode exchange between the TE_1_ mode and TE_2_ mode in the multimode bus waveguide.

**Figure 7:**
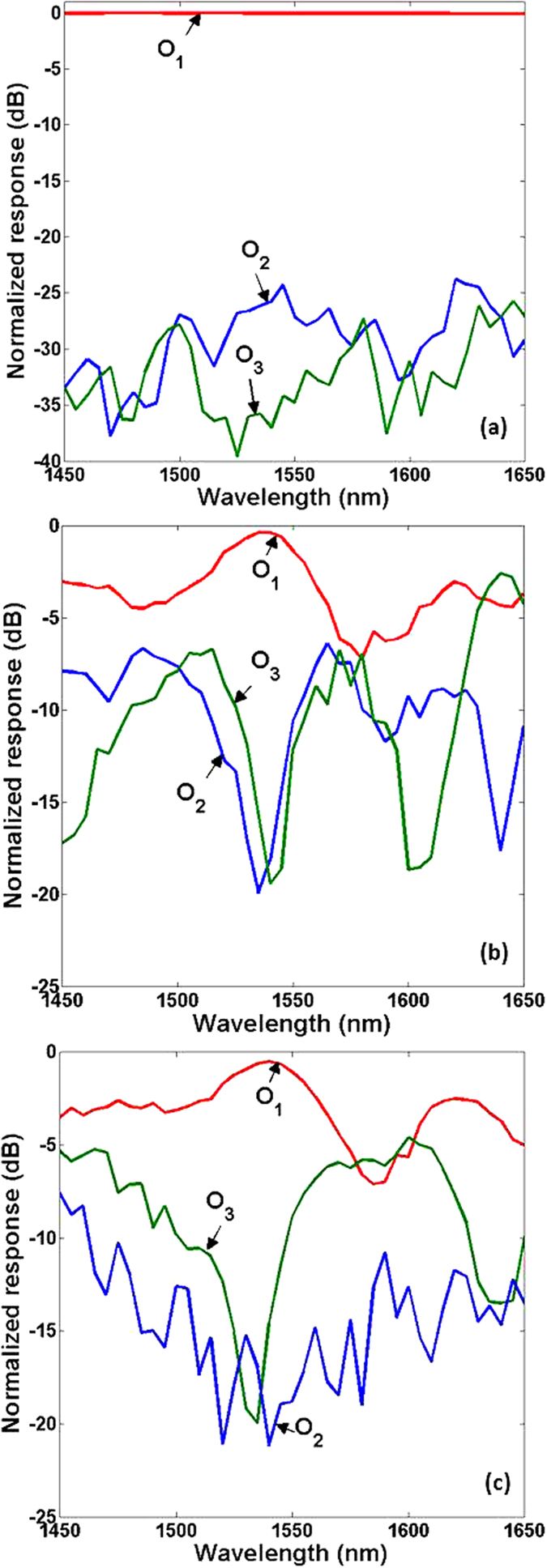
Normalized responses at output ports (O_1_, O_2_ and O_3_) when light is launched into input port (**a**) I_1_, (**b**) I_2_ and (**c**) I_3_.

Figure 7(a–c) show the normalized transmission responses at the three output ports O_1_, O_2_ and O_3_, in which the light is launched into the input ports I_1_, I_2_ and I_3_, respectively. Note that output ports O_1_, O_2_ and O_3_ correspond to the total three modes (TE_0_, TE_1_, TE_2_) after mode exchange between TE_1_ and TE_2_, the residual TE_0_ mode during the TE_1_-TE_0_-TE_2_ process (mode conversion from TE_1_ to TE_2_), and the residual TE_0_ mode during the TE_2_-TE_0_-TE_1_ process (mode conversion from TE_2_ to TE_1_), respectively. It can be seen that O_1_ always has the maximum output response among the three output ports. The excess loss is less than 0.5 dB, showing efficient operation of mode exchange. Meanwhile, the extinction ratio defined by 10·log_10_(*P*_*1*_ /*P*_*i*_) (*P*_*1*_ and *P*_*i*_ are normalized response at output port O_1_ and O_i_, i = 2, 3) is assessed to be larger than 15 dB within a 10 nm wavelength range from 1535 to 1545 nm. The obtained results shown in Figs 5, 6, 7 indicate favorable operation performance of efficient optical mode exchange, which might find interesting applications in robust on-chip network management by exploiting the spatial mode dimension.

## Discussion

In summary, we have proposed on-chip optical mode exchange on SOI platform. The device is based on tapered directional couplers and has a relatively large fabrication error tolerance. The fabrication of the device could be easily realized by single step electron beam lithography followed by inductively coupled plasma etching. The obtained simulation results show effective mode excitation and efficient mode exchange between TE_1_ and TE_2_ modes. A low excess loss less than 0.5 dB and a high extinction ratio larger than 15 dB over a 10 nm wavelength range from 1535 to 1545 nm are achieved. With the obtained results, we believe that optical data exchange in the mode domain could be further realized when each mode carries different data information. The proposed optical mode exchange might facilitate flexible optical data processing functions in an on-chip mode multiplexing systems.

## Method

The mode properties (mode distribution and effective refractive index) of the guided modes in the silicon nanowire are calculated by using finite-element method (FEM) with COMSOL^TM^. The scattering bound condition is considered and the simulation domain is surrounded by rectangular perfectly matched layer (PML). The light propagation is simulated by a three dimensional finite difference time domain (3D FDTD) method.

## Additional Information

**How to cite this article**: Zhang, Z. *et al.* On-chip optical mode exchange using tapered directional coupler. *Sci. Rep.* **5**, 16072; doi: 10.1038/srep16072 (2015).
